# La réponse aux besoins affectifs et cognitifs de l’enfant : Application du modèle cumulatif à la population générale

**DOI:** 10.1177/07067437211020597

**Published:** 2021-06-10

**Authors:** Camille Bandola, Marie-Ève Clément, Annie Bérubé

**Affiliations:** 1 Candidate au doctorat en psychologie clinique Université du Québec en Outaouais; 2 Professeure au Département de psychoéducation et de psychologie et titulaire de la *Chaire de recherche du Canada sur la violence faite aux enfants* Université du Québec en Outaouais; 3 Professeure au Département de psychoéducation et de psychologie Université du Québec en Outaouais

**Keywords:** Négligence, Besoins de l’enfant, Famille, Facteurs de risque, Risque cumulatif, Child neglect, Children’s needs, Family, Risk Factor, Cumulative risk, General population

## Abstract

**Objectifs:**

Plusieurs facteurs de risque sont associés à des conduites à caractère négligent. Leur effet cumulatif, soit l’accumulation du risque indépendamment de la présence ou de l’absence de facteurs spécifiques, est toutefois méconnu. La présente étude a pour objectif de déterminer si le cumul prédit la réponse aux besoins affectifs et cognitifs de l’enfant dans la population générale et d’examiner la présence d’un seuil critique.

**Méthode:**

Un total de 1 102 figures maternelles ayant des enfants âgés de 5 à 9 ans vivant au Québec ont été questionnées par le biais d’un sondage téléphonique. La réponse aux besoins affectifs et cognitifs de l’enfant a été mesurée à l’aide d’une adaptation validée de l’échelle multidimensionnelle des conduites de négligence. Dix facteurs de risque individuels, familiaux et socioéconomiques ont été combinés afin de calculer un indice de risque cumulatif.

**Résultats:**

Les résultats montrent que l’indice cumulatif prédit la réponse aux besoins affectifs et cognitifs de l’enfant dans la population générale. Cet effet est observé pour les familles présentant au minimum deux facteurs de risque et augmente de manière importante lors d’une exposition à cinq facteurs.

**Conclusions:**

La présente étude appuie l’hypothèse du cumul, qui avait jusqu’à présent, principalement été examinée au sein d’échantillons vulnérables ou cliniques. Elle favorise une meilleure compréhension statistique des contextes qui rendent difficiles la réponse de l’environnement de l’enfant d’âge scolaire à ses besoins affectifs et cognitifs dans la population générale. Cette avancée est d’autant plus importante considérant les défis liés à l’identification des enfants à risque de voir leurs besoins négligés.

## Abstract (en)

**Objectives:**

Several risk factors are associated with neglectful behaviors. Yet their cumulative effect, which refers to the accumulation of risk regardless of the presence or absence of specific factors, remains unknown. This study aims to determine whether risk accumulation predicts caregivers’ responses to children’s emotional and cognitive needs in the general population and to examine the presence of clinical thresholds.

**Method:**

A total of 1102 maternal figures of children aged 5 to 9 years old living in Quebec were questioned through a telephone survey. The response to children’s emotional and cognitive needs was measured using a validated version of the Parent-Report Multidimensional Neglectful Behavior Scale. Ten individual, family and socioeconomic risk factors were combined to compute a cumulative risk index.

**Results:**

Results indicate that the cumulative index predicts the response to children’s emotional and cognitive needs in the general population. This effect is observed for families exposed to at least two risk factors and it increases considerably when risk exposure reaches 5 factors.

**Conclusions:**

This study supports the cumulative risk hypothesis, which until now had mainly been examined in vulnerable or clinical samples. It fosters a better statistical understanding of contexts compromising an optimal response to school age children’s emotional and cognitive needs in the general population. This breakthrough is particularity important considering the challenges of identifying children at risk of having their needs neglected.

## Introduction

La réponse qu’offre l’environnement de l’enfant à ses besoins est au cœur de son développement optimal^1^. Dans des contextes médicaux, cliniques et légaux nord-américains, une approche dichotomique est utilisée pour évaluer cette réponse aux besoins^2^. Les seuils utilisés varient selon le contexte, les fins visées ainsi que le professionnel qui porte le jugement^2^. En réalité, la réponse qu’offre l’environnement familial aux besoins des enfants relève plutôt d’un continuum, où à un extrême on retrouve une réponse optimale et à l’autre, une réponse insuffisante qui risque d’entrainer des impacts développementaux négatifs^3^. Une réponse incomplète peut alors être reconnue comme une forme de négligence parentale, surtout si elle est récurrente^3^. Au Québec, la loi sur la protection de la jeunesse définit d’ailleurs la négligence par l’incapacité des adultes à la charge d’assurer les soins essentiels à son développement et à sa sécurité^4^.

La négligence est la forme de maltraitance faisant la plus fréquemment l’objet de signalements fondés, représentant 60% de ceux-ci et touchant principalement les enfants âgés entre 6 et 11 ans^5^. Cette tranche d’âge serait également l’une des plus à risque d’y être exposée dans la population générale^6^. Étant caractérisée par des omissions de comportements ou une absence de soins, la négligence est souvent identifiée de manière indirecte, soit par l’observation de ses conséquences sur l’enfant^7^. Ceci soulève l’importance de mieux comprendre ce phénomène dans la population générale afin d’être en mesure de repérer les enfants qui y sont le plus à risque et d’agir de manière préventive.

Parmi les différents types, la négligence d’ordre affective et la négligence d’ordre cognitive seraient les plus prévalentes, comptabilisant plus de la moitié des signalements fondés^8^. La négligence affective réfère à une difficulté de l’entourage à offrir du réconfort à l’enfant et à lui exprimer de l’affection ^9^. À long terme, les enfants qui en sont victimes sont à risque élevé de recevoir un diagnostic de santé mentale lié à la dépression, l’anxiété, l’usage de substances^10,11^ et au stress post-traumatique^10^. Pour sa part, la négligence cognitive réfère à un manque d’occasions d’apprentissage et à une faible implication relative à l’éducation^9^. Alors qu’elle est associée à un risque élevé chez l’enfant de développer des problèmes internalisés et externalisés^12–14^, la négligence cognitive demeure peu étudiée^15^.

Plusieurs études ont documenté le rôle de facteurs de risque associés aux conduites négligentes dont un faible soutien social, la présence de symptômes dépressifs, la consommation d’alcool ainsi que le stress parental élevé^6,16,17^. Certaines caractéristiques sociodémographiques sont également associées à un risque accru de conduites négligentes, notamment le faible niveau d’éducation du parent, la monoparentalité, le jeune âge ou l’âge avancé du parent, le nombre d’enfants élevé dans le ménage ainsi que la perception d’être pauvre^6,16,18^. Les recherches actuelles montrent que chaque facteur considéré individuellement contribue de manière limitée à l’occurrence des conduites négligentes^16^.

Le modèle cumulatif, d’abord proposé par Rutter^19^ ainsi que Sameroff et ses collaborateurs^20^, a été développé pour prendre en considération la co-occurrence des facteurs de risque psychosociaux et pour évaluer ses conséquences sur le développement de l’individu. Ce modèle stipule que l’accumulation de risque, indépendamment des facteurs de risque spécifiques, influence le développement^19,20^. L’addition des facteurs de risque individuels, familiaux et sociaux permet d’obtenir un score cumulatif qui représente le degré de risque auquel l’enfant est exposé^19,20^. Plusieurs études ont permis de statuer que l’effet cumulatif est un prédicteur de problèmes cliniques^19–21^ et de maltraitance chez les enfants^22–26^. Certaines études suggèrent que l’occurrence de la maltraitance s’accroit graduellement en fonction du nombre de facteurs auquel l’enfant est exposé. Plus le nombre de facteurs augmente, plus le risque de maltraitance est grand, révélant un effet linéaire^23,24^. D’autres études ont démontré que la présence de maltraitance augmente significativement à partir d’un certain nombre de facteurs de risque, sous-tendant un effet par seuil^22,25,26^.

Peu d’études se sont toutefois intéressées au risque cumulatif associé au continuum de réponse aux besoins de l’enfant pouvant mener à de la négligence^22^. À notre connaissance, aucune ne s’est pas penchée sur la réponse aux besoins affectifs et cognitifs spécifiquement auprès de la population générale. Ces connaissances s’avèrent essentielles, particulièrement dans un contexte où la science fait état des conséquences à long terme de ces types de négligence^10,11^ ainsi que de la difficulté de repérage^7^.

### Objectifs de l’étude

Cette étude vise à examiner si le risque cumulatif permet de prédire une réponse non-optimale aux besoins affectifs et cognitifs des enfants âgés de 5 à 9 ans dans la population générale. Elle a également pour objectif de vérifier l’effet cumulatif linéaire et de préciser si une augmentation significative peut être notée à partir d’un certain seuil. À notre connaissance, cette étude est l’une des premières à conceptualiser les facteurs de risque associés à une réponse insuffisante aux besoins affectifs et cognitifs de l’enfant selon un modèle cumulatif, et ce auprès de l’une des tranches d’âge les plus à risque d’y être exposée dans la population générale^6^.

## Méthode

### Participants

Cette étude est une analyse secondaire des données populationnelles issues de *l’Enquête sur la violence familiale dans la vie des enfants du Québec* réalisée par l’Institut de la Statistique du Québec (ISQ) en 2012^27^. L’échantillon est composé de 4 029 femmes québécoises habitant au moins 40% du temps avec un enfant âgé entre 6 mois et 17 ans. Les participantes ont été sélectionnées aléatoirement à partir de la liste des familles éligibles au programme d’Allocation famille du gouvernement du Québec, une aide financière versée aux familles ayant un enfant mineur^28^. Un seul enfant était sélectionné au hasard dans le ménage. Les enfants demeurant dans un logement collectif ainsi que ceux résidant sur une réserve indienne ou sur les territoires cris et Inuits ne font pas partie de la population visée. Un sous-échantillon a été créé pour la présente étude en conservant uniquement les 1 102 figures maternelles ayant un enfant âgé de 5 à 9 ans. Cette recherche a été approuvée par les comités éthiques de l’université d’appartenance des auteurs et de l’ISQ.

Les figures maternelles se retrouvent majoritairement dans la catégorie 35 à 39 ans (57,1%) alors que 14,3 % se retrouvent respectivement dans les catégories 25 à 29 ans, 30 à 34 ans et 40 à 44 ans. La majorité rapporte que la langue parlée à la maison est le français et/ou l’anglais (91,9 %). Plus des quatre cinquièmes (81,2%) des figures maternelles occupent un emploi rémunéré au moment du sondage. L’âge moyen des enfants sélectionnés est de 6,89 ans (ET=1,42). Ils sont issus de familles biparentales (76,3%), monoparentales (14,6%) et recomposées (8,4%).

### Procédure

La collecte de données a été réalisée par le biais d’un sondage téléphonique. Chaque répondante a été questionnée sur la réponse aux besoins affectifs et cognitifs de l’enfant ainsi que sur dix facteurs de risque individuels, familiaux et sociodémographiques associés à une réponse difficile aux besoins de l’enfant^6,16–18^.

### Fidélité des instruments de mesure

Afin de mesurer la fidélité des instruments de mesure, soit le degré auquel ils reflètent le concept étudié^29^, nous avons calculé le coefficient oméga (ω) pour chacun d’entre eux à l’aide du logiciel *R (version 3.6.2)* ^30^. Cette estimation de la fidélité s’échelonne de 0 à 1, où plus la valeur s’approche de 1, plus l’instrument est considéré comme étant fidèle^31,32^.

### Mesure de la réponse non-optimale aux besoins affectifs et cognitifs de l’enfant

La sous-échelle « affective et cognitive » de l’*Échelle multidimensionnelle des conduites de négligence parentale,* composée de quatre items a été utilisée pour documenter la réponse aux besoins des enfants^33,34^. Les dimensions affective et cognitive ont été regroupées puisque qu’une étude de validation de l’outil démontre que les deux construits sont associés^33^. Les questions, qui sont validées pour la tranche d’âge de 5 à 9 ans, font référence aux douze derniers mois et concernent les conduites de l’ensemble des adultes de la maison (Tableau 1)^33^. Les choix de réponses se situent sur une échelle de type Likert (1 = jamais à 4 = la plupart du temps ou toujours). Un score total est calculé à partir de la moyenne des réponses aux quatre items. Considérant la distribution fortement aplatie et asymétrique de l’indice (K = 14,42, ET = 0,15; Sk= 3,33, ET = 0,07) qui enfreint les postulats de normalité, la mesure a été dichotomisée en utilisant un point de coupure en centile tel que proposé par Begle et ses collaborateurs^35^. Les mesures de conduites négligentes requièrent d’ailleurs une adaptation du point de coupure lorsqu’utilisées auprès de la population générale^36^. Spécifiquement, le 80^e^ centile a été utilisé comme seuil à partir duquel la réponse aux besoins de l’enfant est considérée non-optimale par rapport à l’échantillon. Ce point de coupure est utilisé pour divers instruments de mesure afin d’identifier les individus ou familles à risque dans la population générale^27,37^. Dans l’échantillon, ce point de coupure permet de discriminer deux groupes distincts, soit les répondantes dont la réponse aux besoins de leur enfant est optimale (score total de 4) et celles pour qui cette réponse est non-optimale (score total < 4). L’utilisation de ce point de coupure plus libéral ne permet pas d’identifier les conduites négligentes qui feraient l’objet d’un signalement, mais reflète plutôt une réponse non-optimale aux besoins affectifs et cognitifs de l’enfant. Le coefficient oméga de l’échantillon est de 0.68, soit une valeur anticipée^9,34,36^ et similaire aux indices de fidélité de plusieurs instruments de mesure utilisés dans le domaine^38,39^.

**Tableau 1.** Items tirés de l’Indice des conduites à caractère négligent

Au cours de la dernière année, est-il arrivé qu’un adulte de la maison…	
1.	dise à [nom de l’enfant] qu’il l’aime ?
2.	affiche, montre ou accorde de l’importance aux dessins de [nom de l’enfant]?
3.	a témoigné de l’intérêt pour les activités, les jeux ou les passe-temps de [nom de l’enfant]?
4.	aide [nom de l’enfant] à faire ses travaux scolaires ?

### Facteurs de risque

#### Stress lié à la conciliation des obligations familiales et extrafamiliales

Une adaptation du *Job-Family Role Strain Scale*, composée de quatre items a été utilisée^40^. Les choix de réponses se situent sur une échelle de type Likert (1 = jamais à 5 = toujours). La somme des items a été dichotomisée en utilisant le 80^e^ centile comme point de coupure à partir duquel le niveau de stress est considéré comme élevé par rapport à l’échantillon^6,27,40^. La valeur du coefficient oméga pour l’échantillon est de 0.68.

#### Symptômes dépressifs

Une version abrégée du *Center for Epidemiological Studies Depression* (CES-D), comportant 12 items, a été utilisée^41,42^. Les choix de réponses se situent sur une échelle de type Likert (1= jamais à 4 = la plupart du temps ou toujours). Un score sommatif de ≥ 13 a été utilisé comme seuil à partir duquel les symptômes dépressifs sont considérés modérés ou élevés tel que suggéré dans plusieurs études^43,44^. La valeur du coefficient oméga pour l’échantillon est de 0.85.

#### Soutien social

Cinq questions tirées de l’*Échelle de provisions sociales* ont été utilisées^45^. Les choix de réponse s’échelonnent de 1 (fortement en accord) à 4 (fortement en désaccord). Un point de coupure au 80^e^ centile sur la somme des items a été utilisé comme seuil à partir duquel le soutien social est considéré faible par rapport à l’échantillon^27,37^. La valeur du coefficient oméga pour l’échantillon est de 0.78.

#### Consommation d’alcool

Le *Alcohol Use Disorders Identification Test* (AUDIT) composé de 10 items a été utilisé^46^. Les choix de réponse pour la plupart des questions font appel à la fréquence de la situation (1 = jamais à 5 = tous les jours ou presque). Un point de coupure de 8 sur la somme des items a été utilisé afin de statuer sur la consommation à risque^47^. La valeur du coefficient oméga pour l’échantillon est de 0.79.

#### Stress engendré par le tempérament de l’enfant

La sous-échelle « enfant difficile » de la version abrégée de l’*Indice de stress parental* a été utilisée^48^. Elle est composée de 5 énoncés faisant référence au tempérament de l’enfant. Les choix de réponse s’échelonnent sur une échelle de type Likert (1 = fortement d’accord à 4 = fortement en désaccord). Un point de coupure au 80^e^ centile sur la somme des items a été utilisé comme seuil à partir duquel le stress parental est considéré comme élevé par rapport à l’échantillon^37^. La valeur du coefficient oméga pour l’échantillon est de 0.81.

#### Caractéristiques sociodémographiques et socioéconomiques

Sur la base d’études antérieures^22,24^, les caractéristiques suivantes ont été considérées comme des facteurs de risque : diplôme d’études secondaires ou moins, monoparentalité, jeune âge ou âge avancé du parent à la naissance de l’enfant (avant 19 ans et après 34 ans), présence de 3 enfants mineurs ou plus dans le ménage et perception d’être pauvre ou très pauvre.

### Indice cumulatif

Chacun des facteurs de risque a été dichotomisé (0 = absence de risque et 1 = présence de risque) en utilisant les points de coupure prescrits. Suite à cette dichotomisation, un indice cumulatif s’échelonnant de 0 à 10 a été créé^25,49^. Pour chaque répondante, l’indice de risque cumulatif est calculé en additionnant la somme des facteurs de risque présents. Le score obtenu correspond ainsi au nombre de facteurs de risque auquel l’enfant de la répondante est exposé.

### Analyses des données

L’ensemble des analyses a été réalisé en utilisant le logiciel SPSS Statistics (version 25)^50^. Dans le cadre des analyses, l’indice de risque a été traité de deux manières différentes, soit en tant que variable continue et catégorielle. Une première régression logistique binaire a été réalisée afin d’évaluer l’association linéaire entre l’indice de risque continu et la probabilité de présenter une réponse non-optimale aux besoins affectifs et cognitifs de l’enfant. Une seconde régression logistique binaire a ensuite été réalisée afin d’examiner si le risque augmente de manière significative à partir d’un certain score à l’indice cumulatif (modèle par seuil). Pour ce modèle, l’indice cumulatif a été considéré comme une variable catégorielle, où les catégories représentent le nombre de facteurs de risque présents. Un score de 0 à l’indice de risque cumulatif a été utilisé comme niveau de référence pour la variable explicative.

## Résultats

### Facteurs de risque individuels et indice de risque cumulatif

Les données descriptives des dix facteurs inclus dans l’indice de risque cumulatif sont présentées au Tableau 2. Pour l’ensemble des participants, la moyenne obtenue à l’indice de risque cumulatif est de 2,01 (ET = 1,39). Les valeurs obtenues à l’indice cumulatif sont présentées au Tableau 3.

**Tableau 2.** Présence des facteurs de risque inclus dans l’indice de risque cumulatif

Facteurs de risque		Absence de risque (0)		Présence de risque (1)	
		%	n	%	n
1.	Stress élevé lié à la conciliation des obligations familiales et extrafamiliales	63,7	691	36,3	393
2.	Symptômes dépressifs	94,8	1009	5,2	55
3.	Faible soutien social	84,5	920	15,5	169
4.	Consommation d’alcool à risque	87,1	948	12,9	141
5.	Stress élevé engendré par le tempérament de l’enfant	65,2	715	34,8	381
6.	Faible niveau de scolarité	83,7	898	16,3	175
7.	Monoparentalité	85,4	922	14,6	158
8.	Jeune âge ou âge avancé de la mère à la naissance de l’enfant	79,7	858	20,3	219
9.	Nombre élevé d’enfants mineurs dans le ménage	62,4	688	37,6	414
10.	Perception d’une situation financière insuffisante	92,8	999	7,2	77

**Tableau 3.** Présence du nombre de facteurs de risque

Score à l’indice cumulatif de risque	Présence	
	%	n
0	13,3	138
1	25,9	269
2	28,2	293
3	18,8	196
4	9,2	96
5	3,1	32
6 et plus	1,5	16
Total	100	1102

Tel qu’attendu, des corrélations significatives ont été observées entre certains prédicteurs (Tableau 4). Toutefois, celles-ci sont faibles (*r*= -0,15 – 0,27), indiquant que les facteurs de risque utilisés mesurent des concepts distincts et qu’il y a absence de multicolinéarité^29^.

**Tableau 4.** Matrice de corrélations entre les facteurs de risque

	SO	SD	SS	CA	ST	NS	TF	AN	EM	SF
SO	–	0,27**	0,07*	0,05	0,09**	-0,03	-0,01	0,01	0,07*	0,03
SD		–	0,15**	0,01	0,14**	0,08**	0,11**	0,05	-0,04	0,19**
SS			–	-0,06*	0,03	0,11**	0,05	0,06*	0,01	0,15**
CA				–	0,01	-0,03	0,05	-0,01	-0,05	-0,01
ST					–	0,05	0,02	0,01	0,02	0,12**
NS						–	0,10**	-0,02	0,07*	0,21**
TF							–	0,05	-0,15**	0,16**
AN								–	-0,06	0,05
EM									–	0,05
SF										–

Note. *p < 0,05; ** p < 0,01; SO = Stress lié à la conciliation des obligations familiales et extrafamiliales; SD = Symptômes dépressifs; SS = Soutien social; CA = Consommation d’alcool; ST = Stress engendré par le tempérament de l’enfant; NS = Plus haut niveau de scolarité complété; TF = Type de famille; AN = Âge de la mère à la naissance de l’enfant; EM = Nombre d’enfants mineurs dans le ménage; SF = Perception de la situation financière.

### Réponse non-optimale aux besoins affectifs et cognitifs de l’enfant

Dans l’échantillon, 240 répondantes déclarent une réponse non-optimale aux besoins de l’enfant (≥80^e^ centile). Ceci correspond à 21.79% de l’échantillon total. Les résultats de la première régression logistique binaire, où l’indice cumulatif a été considéré en tant que variable continue, révèlent que l’indice cumulatif est associé positivement et de manière significative à une réponse non-optimale aux besoins affectifs et cognitifs de l’enfant (*O.R.*= 1.21, 95 % *I.C.*= 1.09-1.35, *p* < .001). Le modèle révèle que l’indice cumulatif explique 2 % (*Ra* ^2^) de la variance de la réponse non-optimale (*p* < .001).

La deuxième régression logistique binaire visait à examiner à partir de quel seuil la probabilité de présenter une réponse non-optimale aux besoins affectifs et cognitifs de l’enfant augmente de manière significative. L’indice cumulatif a été considéré comme une variable catégorielle. Le modèle révèle que le score à l’indice de risque cumulatif explique 4 % (*Ra* ^2^) de la variance de la réponse non-optimale aux besoins affectifs et cognitifs de l’enfant (*p* < .001). Les résultats révèlent qu’à partir d’un score de 2 à l’indice cumulatif, la probabilité de présenter une réponse non-optimale augmente significativement (Tableau 5; *O.R.*= 2.20, 95 % *I.C.*= 1.30-3.74, *p* < .05). Une augmentation marquée est également observée au score de 5 à l’indice cumulatif (Tableau 5; *O.R.*= 4.33, 95 % *I.C.*= 1.87-10.03, *p* < .01).

**Tableau 5.** Probabilité de présenter une réponse non-optimale aux besoins affectifs et cognitifs en fonction du score à l’indice de risque cumulatif

Score à l’indice de risque cumulatif	B	Erreur standard	OR	95 % IC pour OR
1	0.11	0.29	1.12	(0.64-1.97)
2	0.79**	0.27	2.20	(1.30-3.74)
3	0.54^a^	0.29	1.71	(0.97-3.02)
4	0.83*	0.33	2.29	(1.21-4.35)
5	0.47**	0.43	4.33	(1.87-10.03)
6 et plus	0.25	0.68	1.29	(0.34-4.90)

Note. Un score de 0 à l’indice de risque cumulatif est utilisé comme niveau de référence pour la variable explicative. 0.02 (Cox & Snell) 0.04 (Nagelkerke). Modèle χ^2^ (6) = 24.92, p < 0.001. *p < 0.05; **p < 0.01; ^a^p < 0.10.

## Discussion

Les résultats suggèrent que dans la population générale, plus les familles font face à des facteurs de risque, moins elles sont aptes à répondre aux besoins de leur enfant de manière optimale. Ce constat concorde avec les résultats d’études précédentes soutenant que le risque cumulatif est un prédicteur du risque de maltraitance, incluant la négligence parentale^22–25^.

Les résultats de la présente étude montrent également un effet cumulatif par seuil, où à partir d’une exposition à 2 facteurs de risque, l’environnement familial de l’enfant semble éprouver une difficulté à répondre de manière optimale à ses besoins émotifs et affectifs. L’ajout de risque dans l’environnement est relié à une difficulté plus grande de répondre adéquatement aux besoins des enfants^24,51^. Dans la présente étude, 60.8% des familles de l’échantillon sont exposées à au moins 2 facteurs de risque, ce qui représente l’adversité à laquelle une majorité de parents font face^23,25,52^. Les familles exposées entre 2 et 4 facteurs de risque sont environ 2 fois plus à risque de présenter une réponse non-optimale aux besoins affectifs et cognitifs de leur enfant. Cette similitude dans les rapports de cotes pour les familles présentant de 2 à 4 facteurs suggère la présence d’un plateau. Notons que pour les familles présentant 3 facteurs de risque, leur risque de présenter une réponse non-optimale augmente de près de deux fois comparativement aux familles n’en présentant aucun bien que ce résultat soit marginalement significatif. Ainsi, lors d’une exposition modérée à des facteurs de risque, la probabilité de présenter une réponse non-optimale demeure relativement similaire, tel que démontré pour la probabilité de maltraitance physique^25^.

Des études précédentes ont identifié que le risque de maltraitance augmente considérablement à partir d’une exposition à 5 ou 6 facteurs^22,25,26^. Les résultats de la présente étude vont également dans ce sens. Ces familles sont 4.33 fois plus à risque de présenter une réponse non-optimale aux besoins affectifs et cognitifs de leur enfant. Une étude récente suggère que la négligence est 5 fois plus probable au sein des familles présentant cinq facteurs de risque comparativement au groupe de familles présentant 0, 1 ou 2 facteurs de risque^22^. Il est intéressant de noter que de tels résultats ont été observés auprès de populations cliniques alors que la présente étude a été menée auprès d’un échantillon populationnel.

La réponse non-optimale aux besoins de l’enfant et la négligence, quoique distinctes, se retrouvent sur un même continuum de réponse aux besoins de l’enfant^3^ et peuvent être mises en parallèle. Tel que mentionné ci-dessus, le seuil à 5 facteurs de risque observé dans la présente étude a été identifié précédemment^22^. Toutefois, le seuil à 2 facteurs de risque n’a pas été observé dans les études de risque cumulatif portant sur la négligence^22^ et la maltraitance^23–26^. Cette différence peut être attribué à l’utilisation d’une variable réponse plus sensible, soit la réponse non-optimale aux besoins de l’enfant auprès d’un échantillon populationnel. Les études précédentes ont plutôt utilisé une variable réponse clinique (ex. négligence, maltraitance) auprès de populations cliniques^24,26^. Il est donc attendu qu’un risque de négligence nécessitant une intervention du directeur de la protection de la jeunesse requière une adversité plus importante.

### Limites

Certaines limites doivent être considérées dans l’interprétation des résultats de cette étude. En suivant la méthodologie du modèle cumulatif, les variables ont été dichotomisées, engendrant ainsi une perte d’information quant à l’intensité d’exposition^52^. Certains de ces facteurs ont également été dichotomisés en utilisant des seuils dépendants de l’échantillon tels le rang centile. Compte tenu l’échantillon populationnel, il est ainsi possible qu’un facteur de risque ait été considéré comme présent pour certains participants alors qu’il aurait pu être considéré comme absent au sein d’un échantillon vulnérable. Il est à noter que la généralisation des résultats devrait être effectuée en tenant en compte de l’échantillon de l’étude, soit des familles d’enfants âgés entre 5 et 9 ans dans la population générale.

De plus, le nombre de participants provenant de familles exposées à six facteurs ou plus a limité la puissance des analyses et la capacité à estimer l’effet pour ce groupe. Rappelons également que la présente étude est une analyse secondaire des données issues d’une enquête populationnelle, offrant ainsi les avantages d’une grande taille d’échantillon, d’une représentativité des parents du Québec et d’une rigueur dans la collecte. Cependant, l’inconvénient d’une analyse secondaire est que les hypothèses sont formulées après la collecte et que la méthode est alors restreinte par les concepts mesurés préalablement.

### Conclusion et implications

Plusieurs chercheurs ont récemment soulevé le manque d’études populationnelles portant sur les difficultés à répondre aux besoins de l’enfant^36,53^. À notre connaissance, la présente étude est la première à montrer la présence d’un effet cumulatif pour la réponse non-optimale aux besoins d’ordre affectif et cognitif chez les enfants d’âge scolaire dans la population générale. Elle appuie l’interdépendance des facteurs familiaux et environnementaux, des besoins développementaux de l’enfant et de la réponse à ceux-ci par l’entourage^54^. Les retombées s’inscrivent dans une lignée préventive, où en combinaison avec d’autres études, elle pourrait mener à l’élaboration d’un outil de repérage basé sur l’indice cumulatif^52^. En documentant le degré d’exposition de risque des familles et l’atteinte de seuils critiques^52^, elle pourrait permettre d’identifier les enfants d’âge scolaire les plus à risque d’obtenir une réponse incomplète à leurs besoins affectifs et cognitifs. Ce type d’outil est d’ailleurs reconnu comme étant essentiel pour prévenir la chronicité des conduites négligentes^55^. Ceci est d’autant plus important considérant l’escalade possible des conduites parentales le long d’un continuum de gravité^37,56^ et l’insuffisance de services spécialisés pour les familles présentant un risque de négligence, sans toutefois atteindre la gravité requise pour une intervention du directeur de la protection de la jeunesse^57^.
